# Quantitative and qualitative assessment of the wear pattern of two attachment systems of dissimilar materials for mandibular implant-retained overdentures: an in-vitro study

**DOI:** 10.1186/s12903-023-03693-6

**Published:** 2023-11-30

**Authors:** Rania E. Ramadan, Faten S. Mohamed, Mervat E. Abd-Ellah

**Affiliations:** Department of Prosthodontics, Faculty of Dentistry, Alexandria University, Champollion Street, Azarita, 002034868066 Alexandria Egypt

**Keywords:** Titach attachment, Titanium-to-titanium interface, Locator R-Tx attachment, Titanium-to-nylon interface, Attachment wear, Implant-assisted overdenture

## Abstract

**Background:**

Attachment material is one of the contributing factors to the degree of wear of the attachment components in mandibular implant-retained overdentures. The purpose of this in vitro study was to compare the wear behavior of 2 different attachment systems of dissimilar materials in mandibular implant-retained overdentures by qualitative and quantitative methods.

**Methods:**

Two attachment systems of different materials were utilized (*n* = 16); Titach (Dental Evolutions Inc, Beverly Hills, CA, USA) with a titanium-to-titanium interface and Locator R-Tx (Zest Anchors Inc, Escondido, CA, USA) with a titanium-to-nylon interface. One thousand cycles of overdenture insertion and removal simulating 1-year clinical use were performed. All matrices were removed from the overdentures and all patrices were unscrewed from the implants for wear assessment quantitively using a stereomicroscope and qualitatively using a scanning electron microscope. Data were analyzed by using an inde*p*endent sample t test.

**Results:**

After cyclic loading, stereomicroscopic findings showed that the Titach group had statistically significant higher wear value than the Locator R-Tx group (*p* < 0.001). Moreover, scanning electron microscopy images showed noticeable abrasion in Titach patrix at the area of highest convexity. However, the Locator R-Tx matrix displayed an apparent tear of rubber inserts.

**Conclusions:**

Titach attachment with the titanium-to-titanium interface revealed more wear than Locator R-Tx attachment with the titanium-to-nylon interface. Thus, the type of attachment material influences the degree of wear of the attachment components.

## Introduction

Implant-retained overdentures with attachments offer edentulous patients a better alternative to conventional complete dentures, as overdentures increase comfort, patient satisfaction, and retention [1–3]. The McGill and York Consensus Statements on overdentures recommend a 2-implant-retained overdenture in the mandibular arch as the standard treatment option for an edentulous mandibular arch, to increase complete denture retention [4, 5].

Numerous considerations, including the amount of retention necessary, the rate of wear, maintenance in the long term, and patient satisfaction, influence the choice of a specific attachment design [6–9]. Several attachment systems include bar, ball and socket, magnet, OT equator, Locator, and Titach attachments.

Due to wear over time, loss of attachment retention is the most frequent issue with implant-retained overdentures [10–13]. According to Lambrechts et al., [14] wear is a complicated phenomenon that is the end consequence of numerous interconnected processes acting together. Different types of wear are found, depending on various forms and methods of contact between materials. These include corrosive wear, abrasive wear, adhesive wear, and fatigue wear [15].

Several factors, including the type of attachment material [13, 16–18], the removal and insertion of prostheses [19], the angulation of implants [20–23], and the existence of parafunctional habits [24], may contribute to the wear and tear of attachment components.

The purpose of this in vitro study was to compare by qualitative and quantitative methods the wear behavior of a Titach attachment with titanium-to-titanium interface and a Locator R-TX attachment with clear nylon inserts in mandibular implant-retained overdentures. The null hypothesis was that after recurring cycles of overdenture removal and insertion, the wear behavior of the Titach and Locator R-TX attachment systems in mandibular implant-retained overdentures would be the same.

## Materials & methods

In this study, Titach attachment (Titach; Dental Evolutions Inc, Beverly Hills, CA, USA) and Locator R-Tx attachment (Zest anchors Inc, Escondido, CA, USA) were used. Both attachment systems had two parts, male and female part, known as “patrix” and “matrix” respectively, which were placed into one another to facilitate fastening. The matrix was integrated into the overdenture fitting surface and the patrix was attached to the implant. Titach patrix was made of titanium, whereas the locator R-Tx patrix was made of titanium carbon nitride coating with a pink aesthetic color. Female parts were fabricated from dissimilar materials. Titach matrix was composed of a titanium cap surrounded by a silicone sleeve, while locator R-Tx matrix was formed of titanium housing with various nylon inserts confined in it of different retention; zero retention insert (gray), low retention insert (blue), medium retention insert (pink) and high retention insert (clear) [20, 25].

A sample size of 16 mandibular implant-retained overdentures with 32 attachments was calculated with a software program (G*Power v3.1.9.2; Heinrich Heine University Düsseldorf) [26] supposing a 95% level of confidence and 80% power. Following the early results, it was deemed that no more attachments were needed because the sample size allowed for the identification of highly statistically significant differences.

A set of ready-made maxillary and mandibular completely edentulous silicone molds was used for pouring of one maxillary stone model and 16 mandibular stone models for fabrication of 16 mandibular complete dentures. The same mandibular mold was used for pouring a mandibular model made of epoxy resin (Ramses Medical Products Factory, Alexandria, Egypt) with a 7.5 mm width at the canine region. The epoxy resin was covered with mucosa simulating material made of flexible polyurethane of 1.5 mm thickness [25]. Maxillary and mandibular trial denture bases made of autopolymerizing acrylic resin (Special Tray Material; Acrostone Co Ltd, Cairo, Egypt) with wax occlusion rims were constructed on the stone models and mounted on a mean value articulator, on which maxillary and mandibular acrylic teeth (Acrostone Plus Double Layer anterior and posterior teeth; Acrostone Co Ltd, Cairo, Egypt) were arranged and adjusted. The intercanine distance in the mandibular arch was 22 mm (each was 11 mm from the midline), which simulates the distance between two natural canines [27].

Sixteen mandibular trial denture bases were constructed on the mounted stone models. The same set size mandibular acrylic teeth (size 22) were arranged on all the trial denture bases utilizing the same mounting while keeping the opposing maxillary trial denture base in place to ensure standardization of all the mandibular implant-retained overdentures. The mandibular trial denture bases were finally processed into heat-polymerized acrylic dentures (Denture Base Material; Acrostone Co Ltd, Cairo, Egypt).

**Fig. 1:**
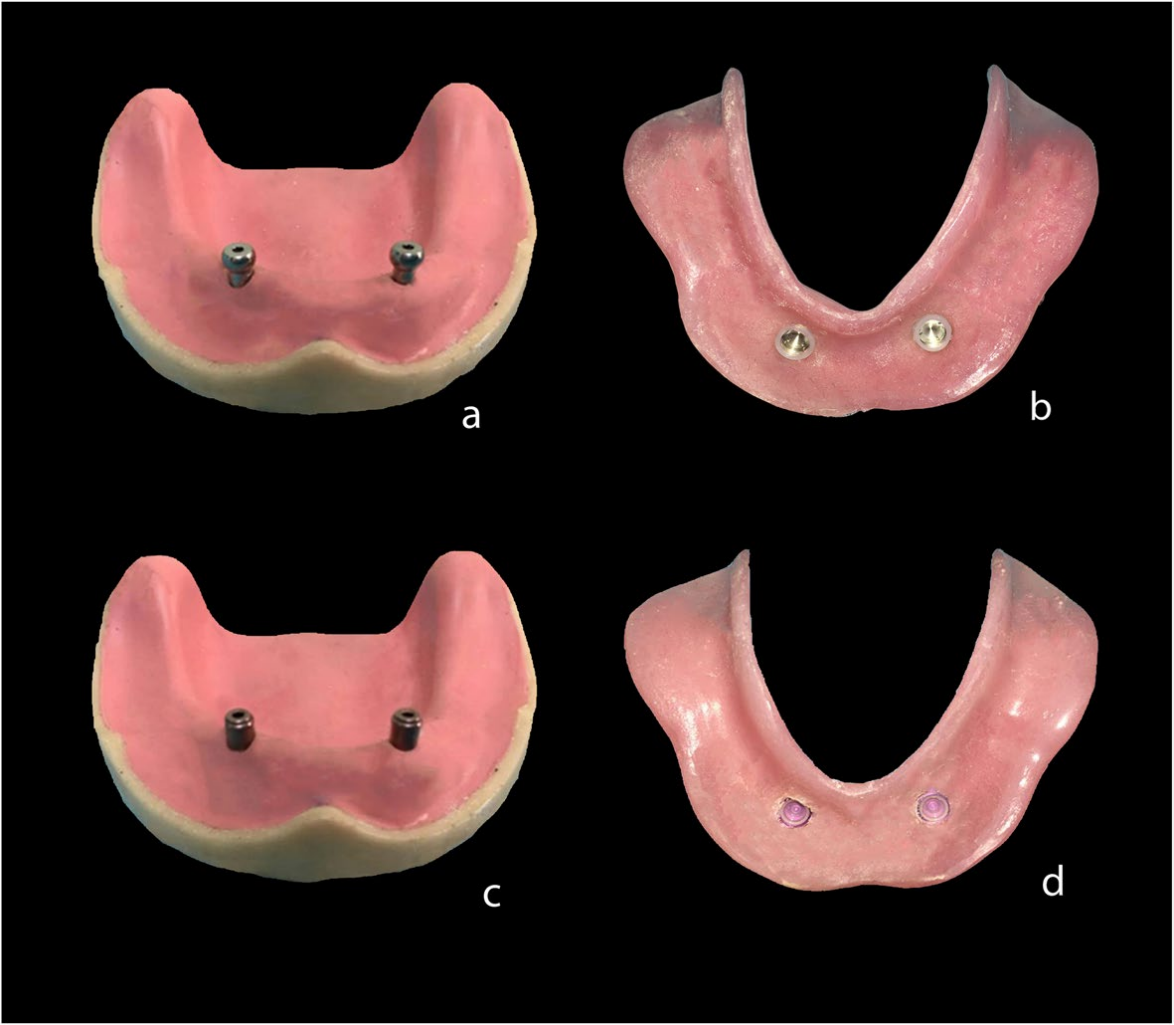
**a** Titach attachments screwed to the implants. **b** Implant-retained overdenture after pick-up of Titach's caps. **c** Locator R-Tx attachments screwed to the implants. **d** Implant-retained overdenture after pick-up of locator R-Tx attachment with clear nylon insert

Two implants (Implanova; Dental Evolutions Inc, Beverly Hills, CA, USA) of length 10 mm and diameter 3.5 mm were placed at the canine area parallel to each other. The 32 attachment caps were directly picked up using autopolymerizing resin. For Titach attachment (Titach; Dental Evolutions Inc, Beverly Hills, CA, USA), the silicone sleeve was placed on the cap during the pick-up, then after setting of the acrylic resin, the protruding part from the cap was cut off by using a sharp scalpel. For locator R-Tx attachment (Zest anchors Inc, Escondido, CA, USA), the black processing insert was used during the pick-up, then substituted with a clear nylon insert (Fig. 1).

**Fig. 2:**
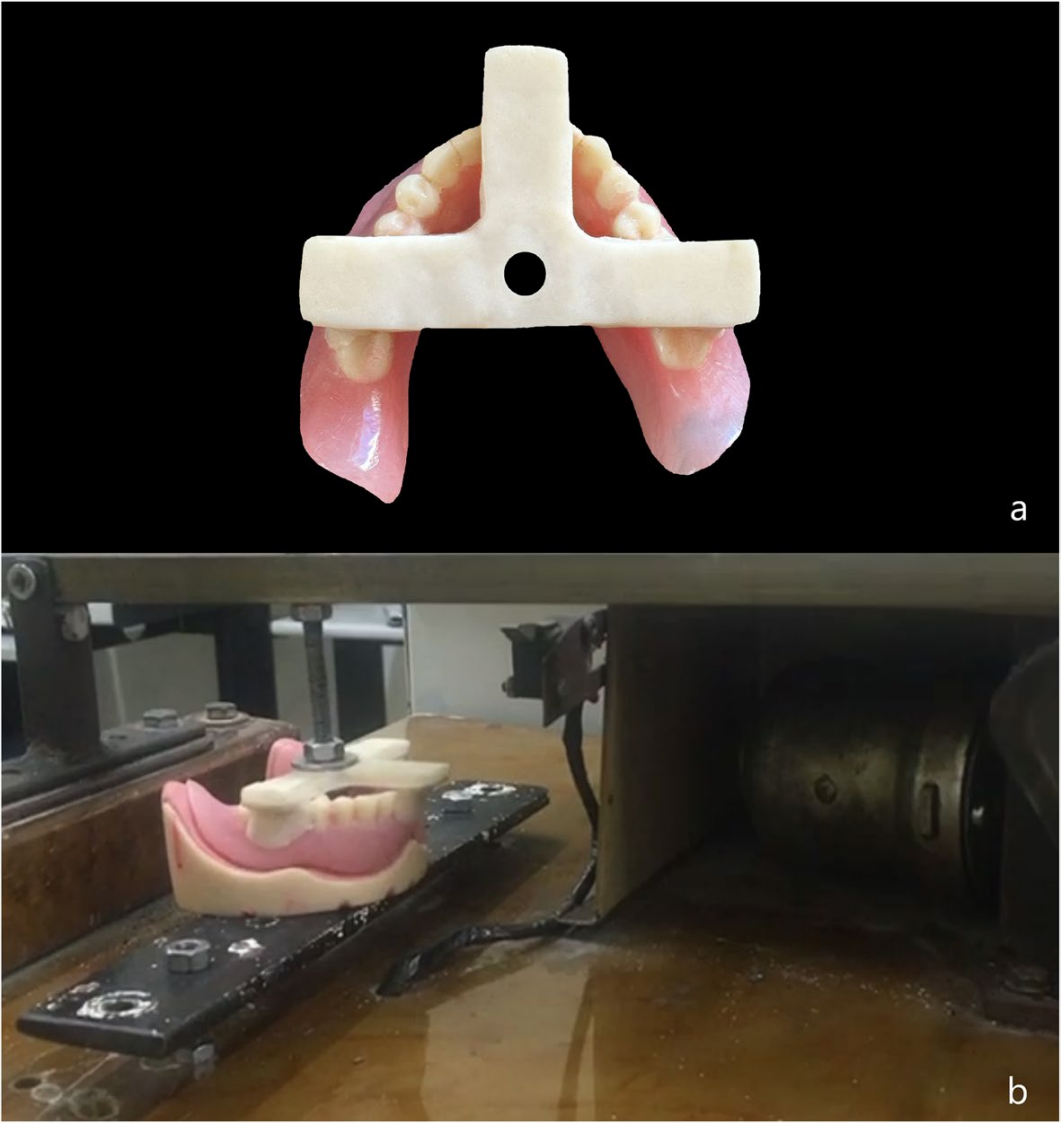
**a** T-shaped acrylic resin plate attached to the occlusal surfaces of the acrylic resin teeth at the incisal and first molar regions of overdentures by using autopolymerizing resin. **b** The center of the T-shaped plate with the overdenture attached to the upper member of the cyclic loading machine

A vertical cyclic tension–compression test was conducted utilizing a custom-made cyclic loading machine simulating overdenture insertion and removal [25]. Every overdenture was subjected to 1000 cycles, corresponding to 1-year clinical use with an average of 3 cycles each day [28, 29]. A T-shaped acrylic resin plate was fabricated to be attached to the occlusal surfaces of the acrylic resin teeth at the incisal and first molar regions of overdentures by using autopolymerizing resin. The center of the T-shaped plate was attached to the upper member of the cyclic loading machine to perform removal and insertion cycles of the overdentures [25] (Fig. 2). Following cyclic loading, all attachment caps (female parts/matrices) were removed from the overdentures using an acrylic resin trimming bur and all male parts/patrices were unscrewed from the implants.

Quantitative analysis of wear patterns of both attachment systems (*n* = 32) was inspected by measuring the inner circumference of the attachment’s matrix as well as the outer circumference of attachment’s patrix under a stereomicroscope (B016, Olympus, Japan; Software: Toup view, version 3.7) at × 25 magnification. According to previous studies [20, 30–34], stereomicroscopy analysis with standardized in vitro conditions exhibited accurate measurements. The measurements were acquired before and after cyclic loading by 2 operators and the mean of the two readings was calculated. Then, the absolute difference between before and after cyclic loading was considered the wear value. In addition, qualitative evaluation of the surface topography of both attachment systems (*n* = 32) was observed by scanning electron microscopy (JSM-IT200; JEOL Ltd, Anhui, China) at different magnifications.

Statistical analysis was performed by using a statistical software program (IBM SPSS Statistics, v23; IBM Corp). The Kolmogorov‒Smirnov test of normality did not show any significance in the distribution of variables; therefore, parametric statistics were used. The test used was an independent sample t test. Furthermore, the interexaminer reliability was calculated by using the intraclass correlation coefficient (ICC) between two operators for each reading. ICC was rated as follows [35]: < 0.40: “poor”, 0.40–0.59: “fair”, 0.60–0.74: “good” and 0.75–1: “excellent”. (α = 0.05).

## Results

**Fig. 3:**
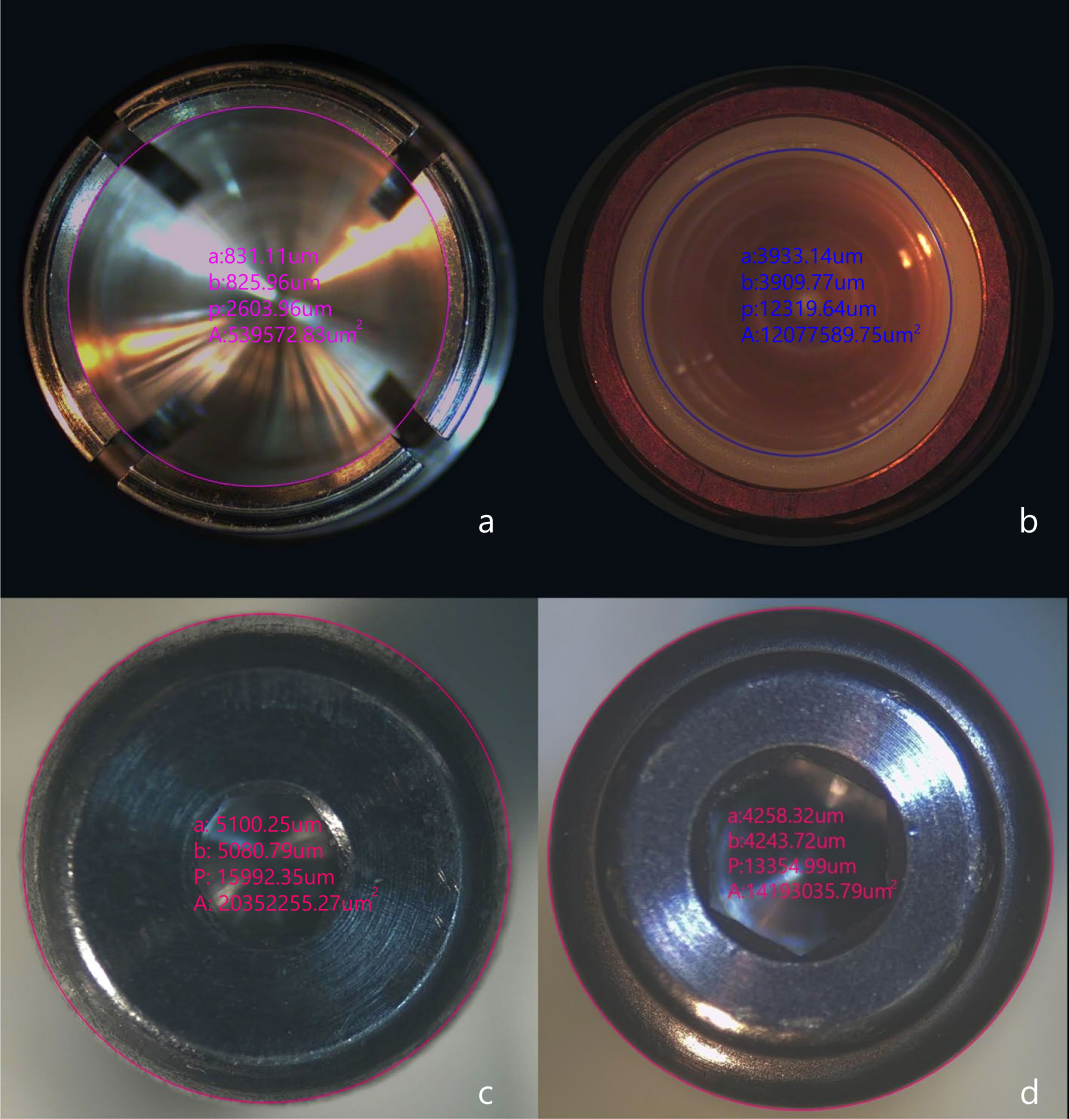
Stereomicroscopy images (original magnification × 25). A software program (Toup view, version 3.7) was used to measure the inner circumference of the attachment matrix and outer circumference of the attachment patrix. **a** Titach matrix. **b** Locator R-Tx matrix. **c** Titach patrix. **d** Locator R-Tx patrix

Regarding the stereomicroscopic measurements, an excellent degree of interexaminer reliability (ICC = 0.99, 95% CI) was found between the two operators. The matrix of the Locator group displayed a statistically significant higher wear value with a mean = 73.72 µm compared with that of the Titach group with a mean = 24.41 µm after cyclic loading (*p* < 0.001). In contrast, the patrix of the Titach group showed a statistically significant higher wear value with a mean = 87.37 µm compared with that of the Locator group with a mean = 1.93 µm after cyclic loading (*p* < 0.001) (Table 1). Moreover, the wear values of matrix and patrix in each group were summed and considered total wear. The Titach group exhibited statistically significant higher total wear value with a mean = 111.79 µm than the Locator group with a mean = 91.68 µm (Table 1, Fig. 3).

**Table 1:** Wear values of the matrix and patrix in the Titach and Locator R-Tx groups

Wear (µm)	Titach attachment group *n* = 16	Locator R-Tx attachment group *n* = 16	*p*
**Matrix**			
- Min–Max	10.69–68.68	47.11–107.77	< 0.001
- Mean ± SD	24.41 ± 14.63	73.72 ± 16.65	
- Standard error of Mean	3.66	4.16	
**Patrix**			
- Min–Max	70.15–99.61	16	< 0.001
- Mean ± SD	87.37 ± 8.27	1.93–5.67	
- Standard error of Mean	2.07	4.31 ± 0.86	
**Total Wear (µm)**			
- Min–Max	85.65–155.76	73.71–104.28	< 0.001
- Mean ± SD	111.79 ± 16.56	91.68 ± 8.56	
- Standard error of Mean	4.14	2.14	

*Min* minimum, *Max* maximum, *SD* standard deviation

**Fig. 4:**
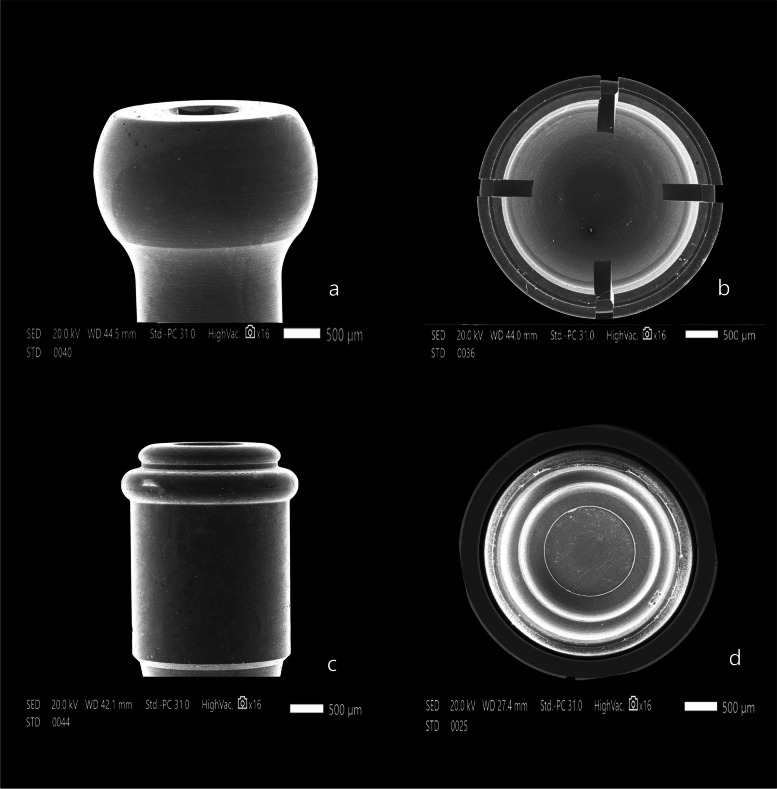
Scanning electron microscope images before cyclic loading (original magnifications × 16), **a** Titach patrix. **b** Titach matrix, **c** Locator R-Tx patrix, **d** Locator R-Tx matrix

**Fig. 5:**
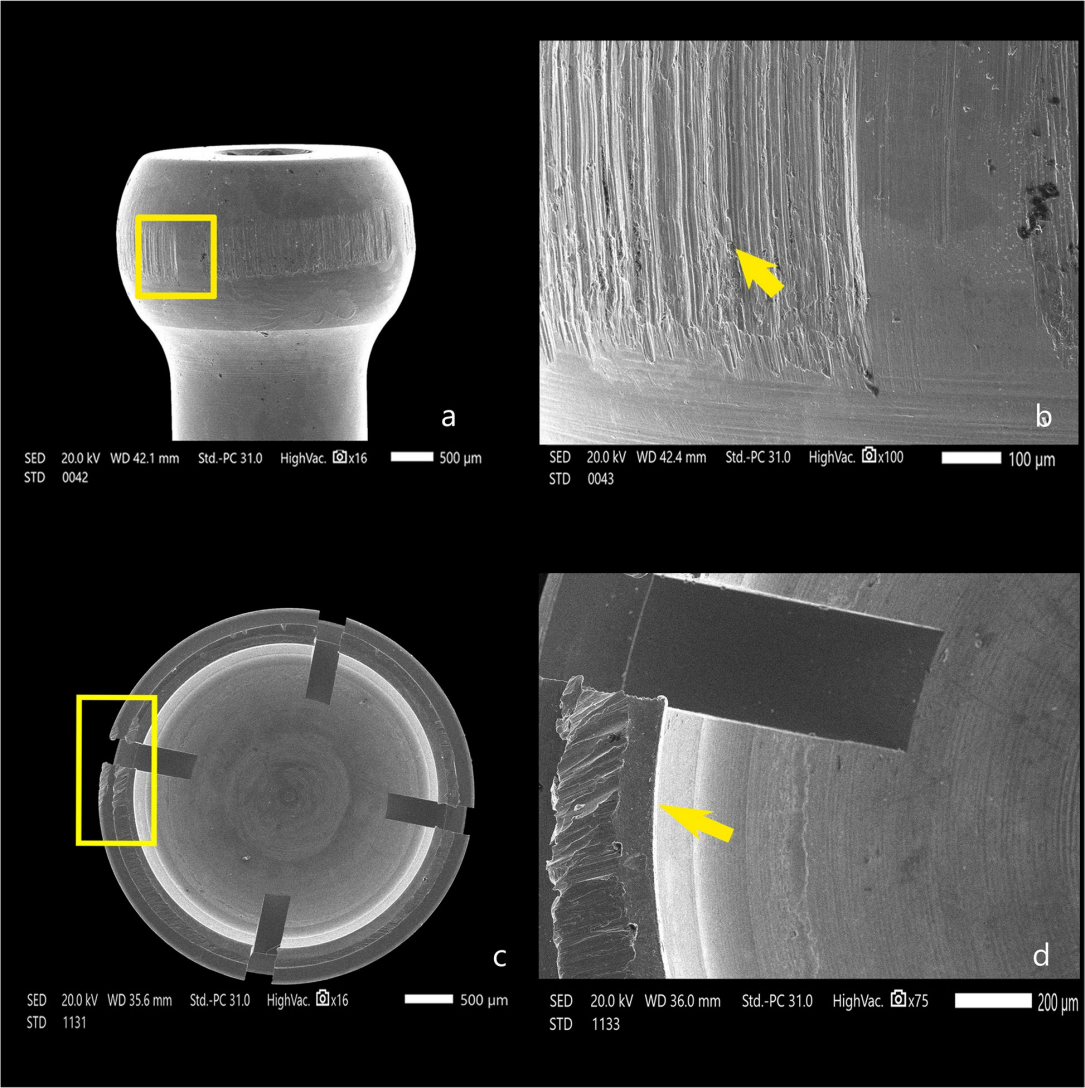
Scanning electron microscope images of Titach attachment after cyclic loading. **A** Titach patrix at × 16 magnification revealing an obvious band of wear and abrasion across the height of contour. **B** Higher magnification (× 100) showing severe wear with metal sloughing. **C** Titach matrix showing a noticeable wear along the inner circumference. **D** Higher magnification (× 75) showing severe wear with metal sloughing

**Fig. 6:**
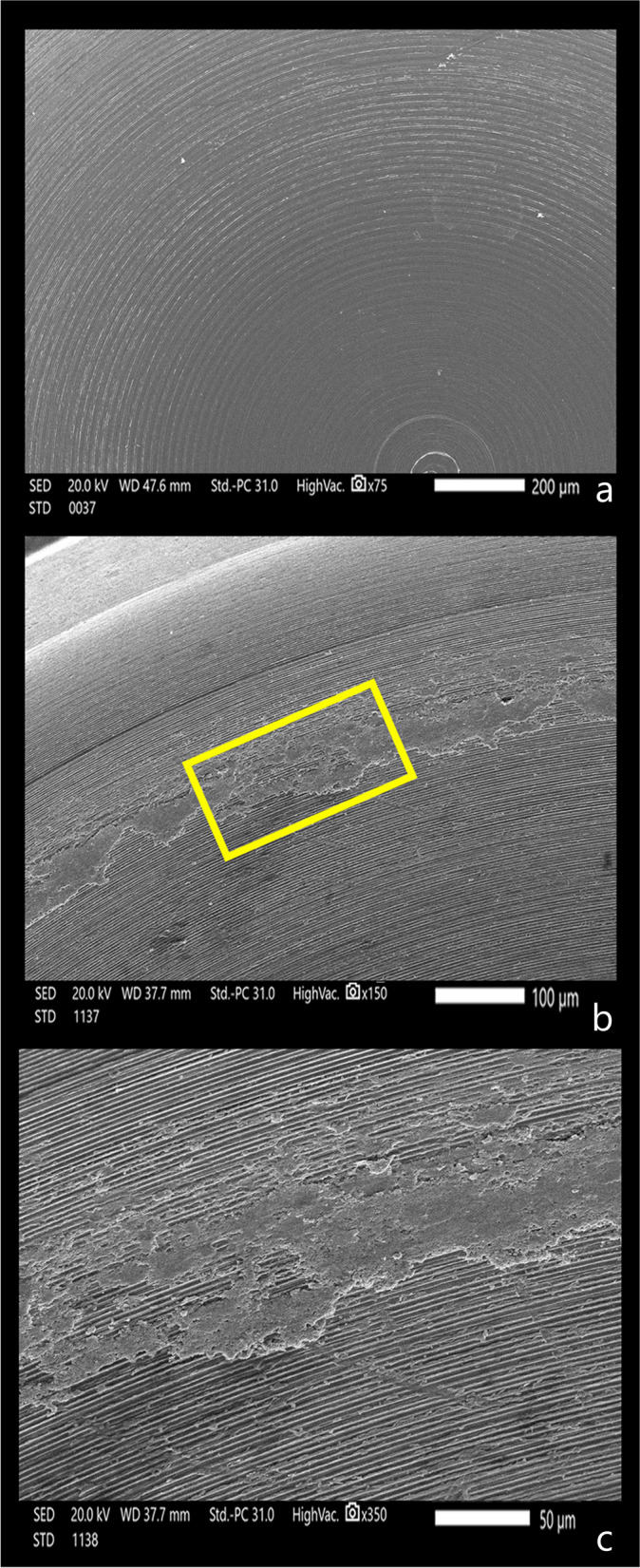
Scanning electron microscope images of the inner surface of the Titach matrix (original magnification × 75, × 150, × 350). **a** Titach matrix before cyclic loading showing regular manufacturing lines. **b**, **c** Titach matrix after cyclic loading revealing accumulation of metallic powder

**Fig. 7:**
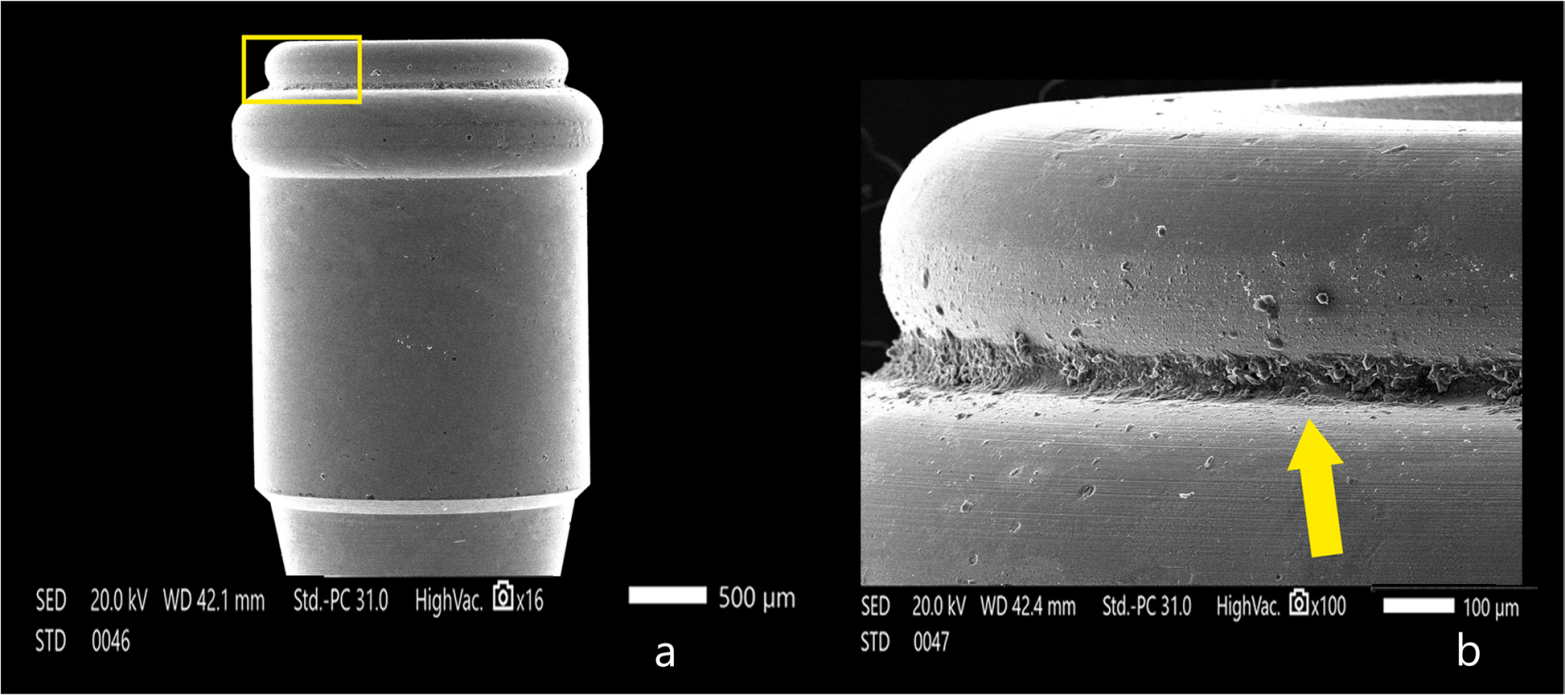
Scanning electron microscope images of Locator R-Tx patrix after cyclic loading showing accumulation of nylon remnants along the narrower coronal area, **A** × 16 magnification, **B** × 100 magnification

**Fig. 8:**
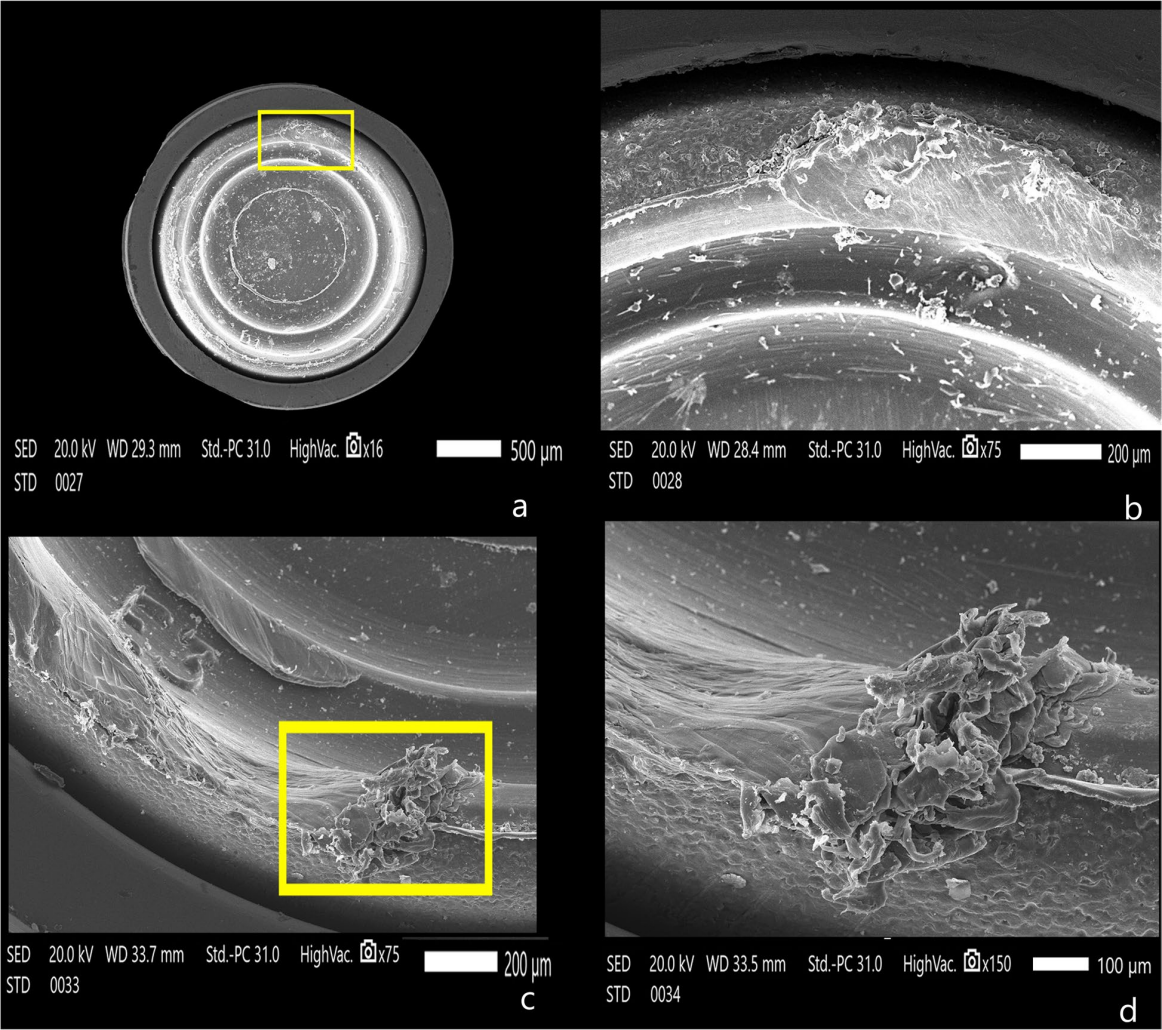
Scanning electron microscope images of the Locator R-Tx matrix after cyclic loading presenting a noticeable tear and deformation along inner circumference, **A** × 16 magnification, **B** × 75 magnification, **C** × 150 magnification

The attachment surfaces of each group were observed by a scanning electron microscope before and after cyclic loading. The Titach patrix presented more noticeable wear changes than the matrix in the form of scratches, abrasion and deformation, especially in the area of highest convexity. Moreover, the inner surface of Titach showed accumulation of metallic powder produced from titanium-to-titanium friction. Furthermore, the patrix of the locator R-Tx group had an accumulation of nylon remnants along the narrower coronal area. However, the matrix of the Locator R-Tx group displayed more apparent wear changes than the patrix in the form of a tear of rubber inserts, surface irregularities and deformations along the inner circumference (Figs. 4, 5, 6, 7 and 8).

## Discussion

The current study investigated 2 attachment designs, Titach and Locator R-Tx, as they were the only compatible attachments with the used implant system. Wear investigation was conducted in an in-vitro study to standardize occlusal loadings away from the absence of other intraoral factors. The present study revealed that the repeated cycles of overdenture removal and insertion significantly have led to different wear behavior in each attachment systems; thus, the null hypothesis was rejected.

Maxillary and mandibular stone casts were mounted on a mean value articulator and acrylic teeth were arranged. The maxillary record base was kept on the articulator while the 16 mandibular record bases were interchanged on the same mounting to preserve the same maxillo-mandibular relation during arrangement of mandibular acrylic teeth to ensure standardization of all the mandibular implant-retained overdentures.

In the epoxy resin edentulous mandibular model, implants were placed parallel to one another based on the suggested implant placement for non-splinted implant-retained overdentures: parallel, in the path of insertion of overdentures, and perpendicular to the occlusal plane to function well [21–23].

Wear frequently occurs within the first 12 months of use, necessitating more frequent maintenance of the attachments [8, 9, 11]. For this reason, the current study was carried out after 1000 cycles of removal and insertion, which correspond to one year of use, to mimic the clinical wear patterns of attachment systems [29, 28].

The type of attachment material influences the degree of wear of the attachment components. Thus, stereomicroscopic findings in the current study reported that the Locator R-Tx rubber matrices manifsted more wear and deformity than the Titach titanium ones. In contrast, the Titach titanium patrices demonstrated more wear than the Locator male ones. This may be attributed to the titanium carbon nitride coating of Locator R-Tx which was more wear resistant [28, 36].

Furthermore, locator R-Tx attachment is made of different materials with different hardnesses; nylon matrix and metal patrix, so most of the wear had occurred in the nylon matrix. This was in accordance with a previous clinical study by Hahnel et al. [37], which showed relevant signs of nylon insert deterioration in clinical use. The nylon inserts are fabricated from polyamide, which is well known to absorb ambient moisture, impairing its mechanical properties and indicating a significant uptake of water of the nylon inserts in comparison to unworn systems. In addition, an in-vitro study by Castrillon et al. [38] reported that frictional wear of locator attachments placed at a 0° position resulted in a decrease of up to 50% in retention of the clear nylon inserts after a simulated 2 years of wear.

Titach attachment is made of titanium matrix and patrix. Titach patrix has a convex form that may have undergone more wear as a result of friction between metal surfaces of the same hardness and frictional coefficient. The results of studies conducted by Yabul et al. [13] Sonbaty et al. [20], Bayer et al. [17], and Branchi et al. [18] investigating metal to metal attachments were similar to those of the present study, showing that the titanium patrix was characterized by the highest wear rate.

Moreover, when evaluating the total wear of each attachment system, the Titach group had undergone more wear than the Locator R-Tx group. This was correlated with a previous study by Ramadan and Mohamed [25] which reported that Titach attachments showed a greater percentage change in the value of retentive force in the form of a reduction in retention (27%) than the Locator R-Tx group (14%). This may be attributed to the fact that wear and deformation can cause loss of retention as the Titach attachment with titanium-to-titanium interface had undergone higher wear, leading to more loss in retentive force values than the resilient nylon insert of the Locator attachment.

However, the wear pattern and surface alteration of the Titach attachment in the current study did not affect its final retentive force after cyclic loading, which was reported in a previous study by Ramadan and Mohamed [25] as higher initial and final retentive forces utilizing Titach attachments compared with utilizing Locator attachments. Nevertheless, implant-retained overdentures with Titach attachments revealed greater loss in retentive force values than Locator attachments after 1-year of mimicked clinical use, and they revealed greater final retentive force values than Locator R-Tx attachments.

Scanning electron microscopy images of Titach patrix demonstrated an obvious band of wear and abrasion across the height of contour. In addition, surface irregularities were noted in the Titach matrices. This may be attributed to the friction caused by the titanium-to-titanium interface. This finding was the same of previous studies [13, 17, 18, 20] which reported that the metal matrix displayed distinct wear patterns due to the same hardness and frictional coefficient of the metallic surfaces when coming into contact with each other. However, scanning electron microscopy images of the Locator R-Tx matrices showed more noticeable wear than the Locator R-Tx patrices in the form of a tear of rubber inserts and surface irregularities. This was consistent with Wichmann et al. [16], who documented that the Locator nylon inserts disclosed recognizable forms of deformation and wear after cyclic loading.

The limitation encountered in the current study was in its in-vitro nature, as it was challenging to precisely replicate the conditions present in the oral cavity. Attachment wear can be influenced by a number of variables, including parafunctional oral habits, temperature, salivary components, dental plaque, and the usage of denture cleansing solutions [15, 28]. Therefore, it is recommended that clinical investigations with long-term follow-up be conducted to verify the findings of the current in vitro study. In addition, volumetric wear quantification of both attachment systems by high-precision industrial scanners is recommended in future studies.

## Conclusions

Considering the limitations of this in vitro study, the following conclusions were drawn:

1. Titach attachment with the titanium-to-titanium interface showed more total wear than Locator R-Tx attachment with the titanium-to-nylon interface after mimicking 1 year of clinical use.
2. Titach attachment presented through scanning electron microscopic findings a distinct pattern of wear and deformation in both matrix and patrix, whereas Locator R-Tx attachment with nylon insert showed identifiable tear signs in the matrix after mimicking 1 year of clinical use.

## Data Availability

The datasets used and/or analyzed during the current study are available from the corresponding author on reasonable request.
